# Relationship between SHARE-FI Frailty Scores and Physical Performance Measures in Older Adult Medicaid Recipients

**DOI:** 10.3390/geriatrics3030051

**Published:** 2018-08-11

**Authors:** Margaret K. Danilovich, Laura Diaz, Daniel M. Corcos, Jody D. Ciolino

**Affiliations:** 1 Department of Physical Therapy and Human Movement Sciences, Northwestern University, Chicago, IL 60201, USA; laura.diaz@northwestern.edu (L.D.); daniel.corcos@northwestern.edu (D.M.C.); 2 Department of Preventive Medicine, Northwestern University, Chicago, IL 60201, USA; jody.ciolino@northwestern.edu

**Keywords:** frailty, SHARE-FI, physical performance measures, home and community-based services

## Abstract

The Survey of Health, Ageing and Retirement in Europe-Frailty Instrument (SHARE-FI) is a frailty assessment tool designed for primary care settings comprised of four self-report questions and grip strength measurement, yet it is not known how SHARE-FI scores relate to objective physical performance measures that assess physical functioning, fall risk, and disability. This cross-sectional, observational study examined the association between SHARE-FI scores and a battery of physical performance measures in a sample of older adult, Medicaid waiver recipients (*n* = 139, mean age = 74.19 ± 8.36 years). We administered the SHARE-FI, Timed Up and Go (TUG), gait speed, and Short Physical Performance Battery (SPPB) in participants’ homes. Among clients, 45% were frail, 35% pre-frail, and 20% non-frail. There were significant differences in all physical performance measure scores with respect to SHARE-FI category. SHARE-FI continuous scores significantly predicted TUG time, all domains of the SPPB, gait speed, and inability to complete the chair rise test. Self-reported walking difficulty and objectively measured gait speed were significantly correlated. The SHARE-FI continuous frailty score predicts scores on a variety of validated physical performance measures. Given the fast administration time, the SHARE-FI could potentially be used to serve as a surrogate for physical performance measures with known association with physical function, fall risk, and disability.

## 1. Introduction

Frailty is a leading cause of dependency among older adults and is correlated with an increased risk for falls, hospitalizations, institutionalization, and death [1,2]. Frailty has been proposed as a syndrome marked by a constellation of biomedical factors that reduce an individual’s capacity to withstand and recover from stressors [3], yet a definitive single operational definition remains undetermined. Various frailty models exist including the Fried phenotype [4] and the Rockwood frailty index or accumulation of deficits model [5]. Lacking a standard definition of frailty, frailty measurement is subsequently challenged. 

While frailty screening is recommended for all persons over the age of 70 [3], health care providers do not routinely perform frailty screening in part due to time constraints [6]. For example, the Fried frailty phenotype requires a complicated physical activity calculation from the Minnesota Leisure Time Activities Questionnaire. Other tools evaluate frailty extensively through self-report data subject to limitations among respondents with cognitive deficits. A further challenge is that the majority of instruments use a categorical scoring system [7] whereby a person is classified as either frail, pre-frail, or not frail. This approach does not capture the fluid nature of frailty in that even within a given frailty category, a person can be more or less frail [5]. Given these limitations, the use of clinically feasible, valid, and reliable tools that assess frailty as both a categorical and continuous outcome is critical to provide assessments that: (1) identify individuals who are pre-frail and frail; (2) are discerning enough to track dynamic changes in frailty, and (3) are discrete enough to respond to therapeutic interventions. 

Originally developed for primary care settings with an administration time of approximately five minutes, the Survey of Health, Ageing and Retirement in Europe-Frailty Instrument (SHARE-FI) [8] evaluates frailty through four self-report questions and one objective measurement of grip strength. Scores on the SHARE-FI are associated with mortality [9,10], hospital readmission following myocardial infarction [11], and longer hospital length of stay [12]. While the short administration time and singular brief physical evaluation (grip strength) are advantageous in the clinical setting, a potential challenge is the reliance upon self-report responses in four of the five frailty constructs (fatigue, appetite, walking difficulties, and physical activity).

A large number of physical performance measures have been validated for older adult populations to evaluate overall physical functioning, as well as specific gait and balance impairments. Diminished physical functioning and frailty are distinct, but related concepts, and to date few studies have compared the relationship between frailty and scores on validated, objectively assessed physical performance measures. Among older adults, there was a significant correlation between frailty and scores on the six-minute walk test, 30-s chair stand test, 30-s arm curl test, chair sit and reach, and grip strength [13]. Among nursing home residents, there were significant differences in Tinetti, Timed Up and Go (TUG), Short Physical Performance Battery (SPPB), and gait speed scores between frail, pre-frail, and non-frail participants [14]. However, no studies have examined the relationship between physical performance measures and SHARE-FI frailty scores. Thus, it is important to understand the relationship between SHARE-FI scores and valid, reliable, and objective measures of physical function that capture components of the frailty phenotype (e.g., slow gait speed and weakness) to clarify if the primarily subjective SHARE-FI could potentially be used as a proxy for these objective measurements. If SHARE-FI could be used as a proxy, it would open up extensive opportunities to screen large groups of individuals and target individuals in need of interventions to reduce the presence of frailty and the associated functional impairments. As such, this study determined the relationship between SHARE-FI scores and scores on objectively assessed, physical performance measures in a sample of older adult, Medicaid waiver recipients.

## 2. Materials and Methods 

### 2.1. Design and Setting 

We employed a cross-sectional study design which was a secondary analysis of an ongoing trial [15]. All participants signed informed consent prior to participation and data were collected in participants’ homes. Despite varied home environments, standardized testing occurred via the use of the same scripted instructions and equipment across all participants. 

### 2.2. Participants 

The study sample included 139 older adults in Illinois receiving the Medicaid Home and Community Based Services (HCBS) waiver through Help at Home, Inc., Chicago, IL, USA. concurrently enrolled in an exercise trial [15] at the time of data collection. The Medicaid HCBS waiver provides long-term care services in a person’s home. In the state of Illinois, HCBS waiver services are offered to individuals who are at least 60 years of age, have net assets less than $17,500 USD, and would otherwise require nursing home services as determined by a case manager. Services offered to beneficiaries include adult day centers that provide health monitoring, medication supervision, personal care, and recreational activities, as well emergency home response services. However, the primary Illinois HCBS waiver service is in-home care provided by a home care aide who assists with non-medical household tasks, laundry, errands, and personal care tasks such as dressing, bathing, and grooming. Participants in this study were English-speaking Help at Home HCBS clients receiving home care aide services, and each individual received $10 USD for study participation. The Northwestern University Institutional Review Board (STU00201777) approved the study protocol prior to initiation.

### 2.3. Measurements

- Participant demographic information included age, race, sex, level of education, housing environment, and living arrangement.
- The SHARE-FI uses the five constructs from Fried’s frailty phenotype: (1) fatigue (termed exhaustion in the Fried criteria), captured as a binary self-report response indicating whether the respondent has too little energy to complete desired tasks; (2) appetite (termed unintentional weight loss in the Fried criteria), indicating self-reported food intake over the last month (diminished, same, or increased); (3) weakness, assessed by two repetitions of grip dynamometry in each hand; (4) walking difficulties (termed gait speed in the Fried criteria), assessed by the self-reported ability to walk 100 m and climb one flight of stairs; and (5) physical activity, measured via self-report of frequency of engagement in activities requiring low to moderate levels of energy [4]. Using a freely available online calculator individualized for both men and women, a scoring algorithm generates a composite frailty score based on these five assessments, and that score categorizes individuals as non-frail, pre-frail, or frail [8]. For females, scores < 0.315 are considered non-frail, scores from 0.316 to 2.130 are pre-frail, and frail scores are values > 2.131. For males, scores < 1.211 indicate non-frail, scores from 1.212 to 3.005 are pre-frail, and frail scores are values > 3.006.
- The five-repetition chair rise test evaluates the length of time it takes an individual to rise from a chair five times, providing a proxy measurement of lower extremity strength. This test has excellent test-retest reliability (*r* = 0.89) and inter-rater reliability (*r* = 0.95) [16]. As a diagnostic tool, completion of five repetitions in more than 15 s is associated with increased risk for falls [16]. Clients completed the five-repetition chair rise using a standard height chair with arms crossed over their chests. Research staff instructed clients to fully stand and sit down five times as fast as possible without using their upper extremities for support.
- The TUG measures the amount of time it takes an individual to rise from a chair, walk to a point 10 feet away, turn, and return to sitting. The TUG is a widely used measure of fall screening among older adults with scores greater than 15 s predicting fall risk at a rate of 87% [5]. The specificity and sensitivity of predicting falls are high with values of 93.3% and 80%, respectively, and the measure has excellent test-retest, inter-rater, and intra-rater reliability [5]. All clients completed one TUG trial using a standard height chair following standardized instructions to complete the test at a comfortable and safe walking speed.
- Argued as the “sixth vital sign”, self-selected gait speed predicts mortality [17,18], hospitalizations [19], health care utilization [20], functional decline [21], and falls [22]. We measured gait speed as the time to complete a three-meter walking distance at a self-selected, comfortable, usual pace with a one-meter acceleration and deceleration length. All clients performed this measure twice with the average speed of the two measures used for analysis.
- The Short Physical Performance Battery (SPPB) [23] is one of the most commonly used physical performance measures for older adults and assesses functional capacity in three conditions: (1) standing balance, (2) walking speed, and (3) chair rise. Total scores range from 0 to 12 and lower scores are associated with hospital re-admission and [24] disability [25]. Scores less than 10 are predictive of all-cause mortality [26].

### 2.4. Statistical Analysis

We summarized participant demographics and assessment scores via descriptive statistics using mean ± standard deviation for continuous variables and frequency/percent for categorical variables. We used kappa statistics with relevant confidence limits to evaluate agreement between assessments and SHARE-FI components. To evaluate relationships between categorical variables (e.g., frailty category and fatigue), we used Chi-squared tests (or Fisher’s Exact tests in the cases of small cell counts). A series of simple linear regression models explored associations between demographics (age, race, sex, etc.) and SHARE-FI scores. We further used linear models to evaluate predictive ability of frailty category as defined by SHARE-FI for physical performance metrics including: gait speed, time to complete five stands, SPPB assessments, and TUG score. We used Tukey’s correction for pairwise comparisons between frailty categories (frail vs. pre-frail, frail vs. non-frail, and pre-frail vs. non-frail). We present Tukey-adjusted *p*-values and confidence limits for these comparisons. All statistical tests assumed a two-sided, 5% level of significance, and we used SAS, version 9.4 (Copyright 2012; The SAS Institute; Cary, NC, USA) for the analyses. 

## 3. Results

### 3.1. Demographics and SHARE-FI Summary Statistics 

The sample was 78% female with an average age of 74 ± 8.4 years and 94% were African-American (Table 1). Only 35% of clients lived in a supportive living environment (e.g., senior living building). With respect to living arrangement, the majority (62%) lived alone. With respect to health, clients reported a median of five comorbidities (range: 1–13) with the most common conditions being visual problems (78%), hypertension (77%), and arthritis (72%). Overall, 45% of clients were classified as frail according to the SHARE-FI, 35% pre-frail, and 20% non-frail. Table 2 shows mean SHARE-FI scores for men and women by frailty category. 

### 3.2. Performance Measures

Table 2 illustrates client performance summary scores on physical measures overall and by frailty category. We detailed gait speeds by 0.1 m/s increments to provide specifics on gait speed variation in the sample using an amount (0.1) that is the meaningful change in gait speed in older adults. We observed a pattern of SHARE-FI results on physical performance measures whereby participants classified as non-frail tended to reflect the best physical functioning scores, pre-frail participants’ tended to score better than the frail group but worse than non-frail group, and frail participants tended to have scores indicative of poorest functioning. For each physical performance measure, with the exception of time to complete five chair stands (*p* = 0.0522), we identified a statistically significant difference in scores across SHARE-FI frailty categories. Sex was not a significant predictor of any physical performance measures with the exception of hand grip dynamometry (*p* < 0.0001). 

### 3.3. SHARE-FI as a Predictor of Physical Performance Measures and SHARE-FI 

Continuous SHARE-FI scores significantly predicted TUG time (*p* = 0.0002), with each one-point increase on SHARE-FI associated with an estimated mean 4.93 s increase (95% CI: 2.42, 7.44 s) in TUG time (Table 3). After adjustment for multiple comparisons, TUG times significantly differed between frail vs. pre-frail (*p* = 0.0104) and frail vs. non-frail (*p* = 0.0003)), but not between pre-frail and non-frail categories (*p* = 0.3383).

The SHARE-FI significantly predicted SPPB scores (overall *p* < 0.0001 for the total score along with each measure). After adjusting for multiple pairwise comparisons, mean total SPPB scores were significantly different for all possible pairwise comparisons (frail vs. pre-frail *p* = 0.0001, frail vs. non-frail *p* < 0.0001, and pre-frail vs. non-frail *p* = 0.0128). Individual subscales (e.g., SPPB Balance Test Score and SPPB Chair Test Score) could not always be distinguished between adjacent frailty categories. 

Whether treated as a continuous (β = −0.06, *p* < 0.0001) or a categorical variable (*p* < 0.0001), SHARE-FI significantly predicted gait speed. Mean gait speed significantly differed between SHARE-FI frailty categories after adjustment for multiple pairwise comparisons. 

Results for the five-repetition sit to stand test are shown for only the 50% of participants able to complete the test following standardized instructions to rise from a chair without using the upper extremities to assist. The SHARE-FI continuous score significantly predicted the inability to complete the five-repetition chair rise test (OR = 1.70, [1.29, 2.25]; *p* = 0.0002). The SHARE-FI category also significantly predicted the inability to complete the five-repetition chair rise (*p* ≤ 0.0001). After adjusting for multiple comparisons, non-frail and pre-frail groups were not significantly different (*p* = 0.1818) 

### 3.4. Relationship between SHARE-FI Self-Report and Gait Speed 

There was variability on the SHARE-FI self-report responses regarding difficulty walking and/or climbing stairs. Agreement on these two items was only moderate (Kappa = 0.50 [95% CI: 0.35, 0.66]). We then created a new variable for the number of difficulties participants self-reported: 0, 1 (i.e., walking or climbing stairs), or 2 (i.e., both walking and climbing stairs) to examine the relationship between the amount of self-reported walking difficulties and gait speed. We calculated a sample spearman correlation coefficient between this new variable and average gait speed that was significant (*p* = 0.0023; *r* = −0.26). Further, linear model results suggested that after adjustment for multiple pairwise comparisons, there was a significant difference in mean gait speed for participants reporting either activity difficulty versus those reporting no difficulties with walking or climbing stairs (*p* = 0.0189). There was also a significant difference between those reporting difficulties with both activities versus those reporting no difficulties (*p* = 0.0007), but no statistically significant difference between participants reporting difficulty with both walking and climbing stairs versus those reporting difficulties with just one activity (*p* = 0.8244).

## 4. Discussion

The major findings from this study provide evidence that: (1) both continuous and categorical SHARE-FI scores significantly predict outcomes on physical performance measures such that frail individuals tend to have the worst physical performance measure scores while non-frail individuals tend to have the best physical performance measure scores; (2) pairwise comparison of SHARE-FI frailty categories finds significant differences between frail and non-frail categories on physical performance measures, while pre-frail and non-frail and pre-frail and frail are less distinguishable in the chair rise, gait speed, TUG, and SPPB balance scores; and (3) while objectively measured gait speed is significantly correlated with both self-reported walking and stair climbing difficulties on the SHARE-FI, this is a small, negative correlation and there is only moderate agreement between self-report walking difficulty and stair climbing difficulty. 

Assessment of physical function is critical for those with frailty. The advantage of physical performance measures is that they provide objective, precise, valid, and reliable ways to describe functioning while overcoming limitations of recall and response bias in self-report measures. However, these objective measures are often inconvenient to use in the clinic setting from a time, equipment, space, and test-training standpoint. Thus, the possibility of replacing physical performance testing with a frailty assessment that accurately predicts physical performance is of clinical importance. Our findings demonstrate that there is a significant difference in physical performance outcomes between different frailty categories as determined on the SHARE-FI. A frailty classification on the SHARE-FI predicts low physical performance measure scores, such that spending additional time administering specifics tests like the SPPB, gait speed, or TUG may be unwarranted in this population unless there is a specific rationale. 

It is interesting to note the marked gait speed impairment in this sample. Even among the non-frail participants, the gait speed was 0.73 m/s which is lower than the 0.8 m/s gait speed threshold that has been used previously to define frailty [27]. Because the SHARE-FI uses a self-report question, “…do you have any difficulties walking 100 m” to evaluate gait speed, it may be that this subjective question evaluates endurance more than actual walking speed. The weak correlation between objectively measured gait speed and self-reported walking difficulties highlights a potential limitation of the SHARE-FI in adequately evaluating the frailty criteria related to walking. As such, supplementing the SHARE-FI with an objective measure of gait speed may be of clinical importance, particularly in light of evidence showing the significant association between gait speed and a variety of clinical conditions and health outcomes [18].

Results from pairwise comparison of SHARE-FI frailty categories showed non-significant findings in the areas of chair rise, gait speed, TUG, and SPPB balance scores when comparing pre-frail to non-frail and pre-frail to frail individuals. Should an older adult be determined as pre-frail on the SHARE-FI, there may be an added benefit to supplementing SHARE-FI with physical performance measures to obtain a more complete assessment of the participant’s lower limb strength and balance. 

While our findings show that individuals who self-report walking difficulties on the SHARE-FI are more likely to have lower gait speed, this is not a particularly strong relationship. Of clients who reported difficulties walking 100 m, 15% reported no difficulty climbing stairs. It might be assumed that since stair climbing is a more challenging task than walking, those with difficulty walking should correspondingly have stair-climbing difficulty. As over one-third of the sample lived in an environment without stairs, it may be that the lack of stair climbing causes an overestimation of their ability such that respondents report no difficulty with stairs. Others have documented that self-reported activities of daily living (ADL) and instrumental ADL (IADL) performance is moderately associated with physical performance. Older, hospitalized patients consistently overrated abilities in both ADL and IADL performance compared with observed ADL and IADL performance [28] and only 13% of older adults correctly rated their IADL performance when compared to objectively-measured IADLs [29]. Taken together, our work highlights the potential limitations of self-reported performance on the SHARE-FI and the potential need for supplementing these questions with gait speed assessment, particularly for patients reporting falls or mobility impairment to provide a more comprehensive assessment of frailty and functioning. 

Limitations of this work include a small, predominantly minority sample from the Chicagoland area. We do not know how results would translate among other populations. We did not collect a cognitive measure on participants; thus, our understanding of the relationship between cognition, self-report, and physical performance is limited. Further work in this area should explore this association. Finally, the SHARE-FI and physical performance measures in this study capture the physical domain of frailty. Other domains of frailty, such as social and psychological, are not evaluated in these tools. To fully capture a holistic view of frailty in older adults, evaluation of multiple domains is necessary. 

Despite these limitations, our findings demonstrate that both continuous SHARE-FI scores and the corresponding frailty categories determined by these scores predict physical performance measure scores. Given the clinical ease of SHARE-FI assessment, this tool could be used to infer performance on established and validated physical performance measures. Should a person score as non-frail or frail on the SHARE-FI, our results would suggest that physical performance measures could be inferred and there might not necessarily be an added value in spending time and resources on taking these additional measurements. However, for pre-frail individuals, administering physical performance measures might be warranted to fully evaluate the extent of functional impairments. Finally, in light of our findings of a weak relationship between self-reported walking difficulties and gait speed, objective measurement of gait speed may be useful in quantifying gait impairment in order to direct additional medical referral and/or treatment. 

## Figures and Tables

**Table 1:** Client Demographics.

Demographic item	Mean (Std Dev)
**Age**	74.19 (8.36)
**Sex (*n* and %)**	
Female	109 (78.42)
Male	30 (21.58)
**Race (*n* and %)**	
Other	9 (6.47)
African American	130 (93.53)
**Education (*n* and %)**	
<High School	35 (25.18)
High School	48 (34.53)
Some College	35 (25.18)
4-year Degree or Higher	21 (15.11)
**Housing Environment**	
House	42 (30.22)
Apt/Townhome/Condo	48 (34.53)
Senior Living Building	49 (35.25)
**Living Arrangement**	
Alone	86 (61.87)
Spouse/Partner	11 (7.91)
Other	42 (30.22)

**Table 2:** Summary Statistics on Physical Performance Measures.

Physical Performance Measure	Overall		Non-Frail		Pre-Frail		Frail		*p*-Value ^
	*n* = 139		*n* = 28 (20.14%)		*n* = 48 (34.53%)		*n* = 63 (45.32%)		
	Mean	Std	Mean	Std	Mean	Std	Mean	Std	
Frailty Score (SHARE-FI) *	1.89	1.44	−0.16	0.71	1.51	0.51	3.1	0.85	<0.0001
**Hand Grip Dynamometry (kg)**	23.96	9.94	31.38	9.93	26.7	8.74	18.59	7.68	<0.0001
Females	21.39	7.65	26.41	6.30	24.66	6.45	17.66	6.95	<0.0001
Males	33.33	11.69	38.00	10.21	34.45	11.99	24.94	9.87	
**Average Gait Speed (m/s)**	0.55	0.26	0.73	0.21	0.56	0.26	0.46	0.23	<0.0001
**Gait Category**	*n*	%	*n*	%	*n*	%	*n*	%	<0.0001
<0.5 m/s	58	41.73	4	14.29	18	37.5	36	57.14	
[0.5–0.6] m/s	21	15.11	5	17.86	4	8.33	12	19.05	
[0.6–0.8] m/s	36	25.9	9	32.14	15	31.25	12	19.05	
[0.8–1.0] m/s	19	13.67	6	21.43	11	22.92	2	3.17	
≥1.0 m/s	5	3.6	4	14.29	0	0	1	1.59	
**Time to Complete 5 Stands (s)**	21.22	8.12	18.79	5.03	20.74	7.44	24.69	10.72	0.0522
**SPPB Test Scores Total Score**	4.78	2.75	6.96	2.43	5.33	2.57	3.38	2.21	<0.0001
**Balance Test Score**	1.99	1.36	2.79	1.1	2.31	1.46	1.4	1.13	<0.0001
**Gait Speed Test Score**	2.09	1.12	2.89	0.92	2.29	1.13	1.57	0.93	<0.0001
**Chair Stand Test Score**	0.71	0.84	1.29	0.98	0.73	0.71	0.43	0.73	<0.0001
**Tug Score (s)**	27.96	20.6	18.22	8.2	24.77	16.05	36.53	25.68	0.0002
**Tug Category**	*n*	%	*n*	%	*n*	%	*n*	%	<0.0001
<15 s	25	17.99	12	42.86	10	20.83	3	4.76	
≥15 s	114	82.01	16	57.14	38	79.17	60	95.24	
**Unable to Complete 5 Stands**	69	49.64	6	21.43	20	41.67	43	68.25	<0.0001

* Score range: −1.17 to 6.33. *p*-Values ^ correspond to either overall linear model Wald test or chi-squared test result (for categorical outcomes).

**Table 3:** Predictive Ability of Survey of Health, Ageing and Retirement in Europe-Frailty Instrument (SHARE-FI) for Physical Performance Measures.

Outcome	Predictor = Continuous SHARE-FI Score			Predictor = Frailty Categories as Determined by SHARE-FI Score					
	1 Point Increase: Increasing Frailty Score			Frail vs. Pre-Frail		Frail vs. Non-Frail		Pre-Frail vs. Non-Frail	
	Estimate (β)	95% CI	*p*-Value	Estimate (95% CI) ^	*p*-Value ^	Estimate (95% CI) ^	*p*-Value ^	Estimate (95% CI) ^	*p*-Value ^
TUG Score (s)	4.93	(2.42, 7.44)	0.0002	11.76 (2.32, 21.20)	0.0104	18.31 (7.42, 29.21)	0.0003	6.56 (−4.47, 17.58)	0.3383
SPPB Balance Test Score	−0.35	(−0.50, −0.20)	<0.0001	−0.92 (−1.48, −0.35)	0.0006	−1.39 (−2.06, −0.72)	<0.0001	−0.47 (−1.18, 0.23)	0.2519
SPPB Gait Speed Test Score	−0.31	(−0.43, −0.19)	<0.0001	−0.72 (−1.17, −0.27)	0.0003	−1.32 (−1.86, −0.78)	<0.0001	−0.60 (−1.16, −0.04)	0.0335
SPPB Chair Test Score	−0.20	(−0.29, −0.11)	<0.0001	−0.30 (−0.65, 0.05)	0.1130	−0.86 (−1.28, −0.44)	<0.0001	−0.56 (−0.19, −0.12)	0.0089
SPPB Total Score	−0.87	(−1.15, −0.58)	<0.0001	−1.95 (−3.03, −0.87)	0.0001	−3.58 (−4.87, −2.30)	<0.0001	−1.63 (−2.97, −0.29)	0.0128
Average Gait Speed (m/s)	−0.06	(−0.09, −0.03)	<0.0001	−0.11 (−0.22, 0.00)	0.0579	−0.27 (−0.40, −0.14)	<0.0001	−0.16 (−0.30, −0.03)	0.0121
Unable to Complete 5 Stands *	1.70	(1.29, 2.25)	0.0002	3.01 (1.18, 7.66)	0.0057	7.88 (2.25, 27.57)	0.0003	0.38 (0.11, 1.37)	0.1818

* Model estimates are OR (95% CI). ^ Tukey-adjusted 95% confidence intervals and *p*-values for multiple pairwise comparisons.
